# Characterization of Convergent Suppression by UCL-2077 (3-(Triphenylmethylaminomethyl)pyridine), Known to Inhibit Slow Afterhyperpolarization, of *erg*-Mediated Potassium Currents and Intermediate-Conductance Calcium-Activated Potassium Channels

**DOI:** 10.3390/ijms21041441

**Published:** 2020-02-20

**Authors:** Hung-Te Hsu, Yi-Ching Lo, Sheng-Nan Wu

**Affiliations:** 1 Department of Anesthesia, Kaohsiung Medical University Hospital, Kaohsiung City 80756, Taiwan; hdhsu1228@hotmail.com; 2 Faculty of Anesthesiology, College of Medicine, Kaohsiung Medical University, Kaohsiung 80708, Taiwan; 3 Department of Pharmacology, Kaohsiung Medical University, Kaohsiung 80708, Taiwan; 4 Department of Physiology, National Cheng Kung University Medical College, Tainan 70101, Taiwan; 5 Department of Medical Research, China Medical University Hospital, China Medical University, Taichung 40402, Taiwan

**Keywords:** UCL-2077, *erg*-mediated potassium current, intermediate-conductance calcium-activated potassium channel, voltage hysteresis, action current, simulation

## Abstract

UCL-2077 (triphenylmethylaminomethyl)pyridine) was previously reported to suppress slow afterhyperpolarization in neurons. However, the information with respect to the effects of UCL-2077 on ionic currents is quite scarce. The addition of UCL-2077 decreased the amplitude of *erg*-mediated K^+^ current (*I*_K(erg)_) together with an increased deactivation rate of the current in pituitary GH_3_ cells. The IC_50_ and *K*_D_ values of UCL-2077-induced inhibition of *I*_K(erg)_ were 4.7 and 5.1 μM, respectively. UCL-2077 (10 μM) distinctly shifted the midpoint in the activation curve of *I*_K(erg)_ to less hyperpolarizing potentials by 17 mV. Its presence decreased the degree of voltage hysteresis for *I*_K(erg)_ elicitation by long-lasting triangular ramp pulse. It also diminished the probability of the opening of intermediate-conductance Ca^2+^-activated K^+^ channels. In cell-attached current recordings, UCL-2077 raised the frequency of action currents. When *KCNH2* mRNA was knocked down, a UCL-2077-mediated increase in AC firing was attenuated. Collectively, the actions elaborated herein conceivably contribute to the perturbating effects of this compound on electrical behaviors of excitable cells.

## 1. Introduction

UCL-2077 (triphenylmethylaminomethyl)pyridine), which was discovered as a small-molecule blocker, has been particularly reported to suppress the slow or late afterhyperpolarizations existing in different regions of either central neurons or brain slices [1,2,3,4,5,6,7,8]. Earlier work has also demonstrated the capability of this compound to perturb the activity of *KCNQ* channels [4]. However, another paper showed that it was unable to suppress the slow afterhyperpolarization recorded from thalamic midline neurons [9]. Whether this compound produces any modifications on different types of membranous ion channels thus remains unresolved.

The *I*_K(erg)_ encoded by three different subfamilies of the gene *KCNH* is known to give rise to the pore-forming α-subunit of *erg*-mediated K^+^ (i.e., K_erg_ or K_V_11) channels [10], which is regarded to constitute the cloned counterpart of the rapidly activating delayed-rectifying K^+^ currents in heart cells. These currents inherently existing in neurons or in different types of electrically excitable cell, such as endocrine or neuroendocrine cells, can highly influence the maintenance of the resting potential as well as the increase in subthreshold excitability [11,12], thereby leading to marked changes in the discharge rate of spontaneous action potentials (APs) [13,14,15,16]. As a consequence, the resultant perturbations in the activity of such K^+^ channels have the propensity to change the stimulus–secretion coupling of these cells [13,17,18,19]. Previous work has also revealed that the activity of K_erg_ channels can be a regulator of tumor cell apoptosis and proliferation [20]. Whether UCL-2077 produces any modifications in this type of ion channel is largely not explored, although it has been demonstrated to suppress slow afterhyperpolarization [2,3,4,5,6,7,8].

The intermediate-conductance Ca^2+^-activated K^+^ (IK_Ca_) channels (also known as K_Ca_3.1, SK4, IKCa1 or *KCNN4*), which are encoded by the *KCNN4* gene, have been increasingly studied. However, the research into these channels has been focused mostly in different non-excitable or neoplastic cells with regard to their potential roles in cell behaviors that include hormonal secretion, cell migration, cell proliferation, and regulation of Ca^2+^ influx, K^+^ efflux, or both [17,20,21,22,23]. Specifically, these channels possess single-channel conductance of 20–60 pS and their pharmacological profiles are distinct from those of large- or small-conductance Ca^2+^-activated K^+^ channels [22,24,25,26].

The modulators of functional IK_Ca_ channels have recently been demonstrated to perturb functional activities in different types of central neurons, such as membrane afterhyperpolarization [3,9,21,26,27,28,29]. Importantly, previous studies have reported the ability of UCL-2077 to disturb the slow afterhyperpolarization found in central neurons, suggesting a possible role in the regulation of Ca^2+^-dependent K^+^ conductance [1,5,9]. Whether the presence of UCL-2077 effects any substantial perturbation in the activity of Ca^2+^-activated K^+^ channels is therefore worthy of being thoroughly explored.

Therefore, in the current study, we wanted to assess whether the presence of UCL-2077 is capable of exerting any modifications on different types of membrane ion current. Findings from the present experimental observations highlight, for the first time, the notion that UCL-2077 can interact with the kinetic gating of K_erg_ channels to suppress the amplitude of *I*_K(erg)_. This compound was effective at suppressing the activity of IK_Ca_ channels, although minimal regulatory action on the open probability of large-conductance Ca^2+^-activated K^+^ (BK_Ca_) channels was seen. It is thus important to mention that the inhibition of both *I*_K(erg)_ and IK_Ca_-channel activity caused by UCL-2077 may potentially converge to act on the functional activities of neurons, and neuroendocrine or endocrine cells (e.g., afterhyperpolarization following repetitive firing) [8,9].

## 2. Results

### 2.1. Effect of UCL-2077 on I_K(erg)_ in Pituitary GH_3_ Cells

**Figure 1:**
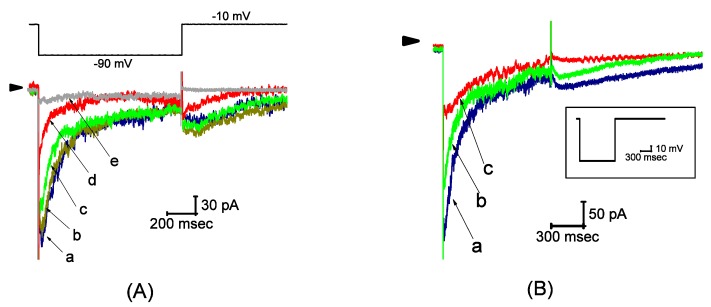

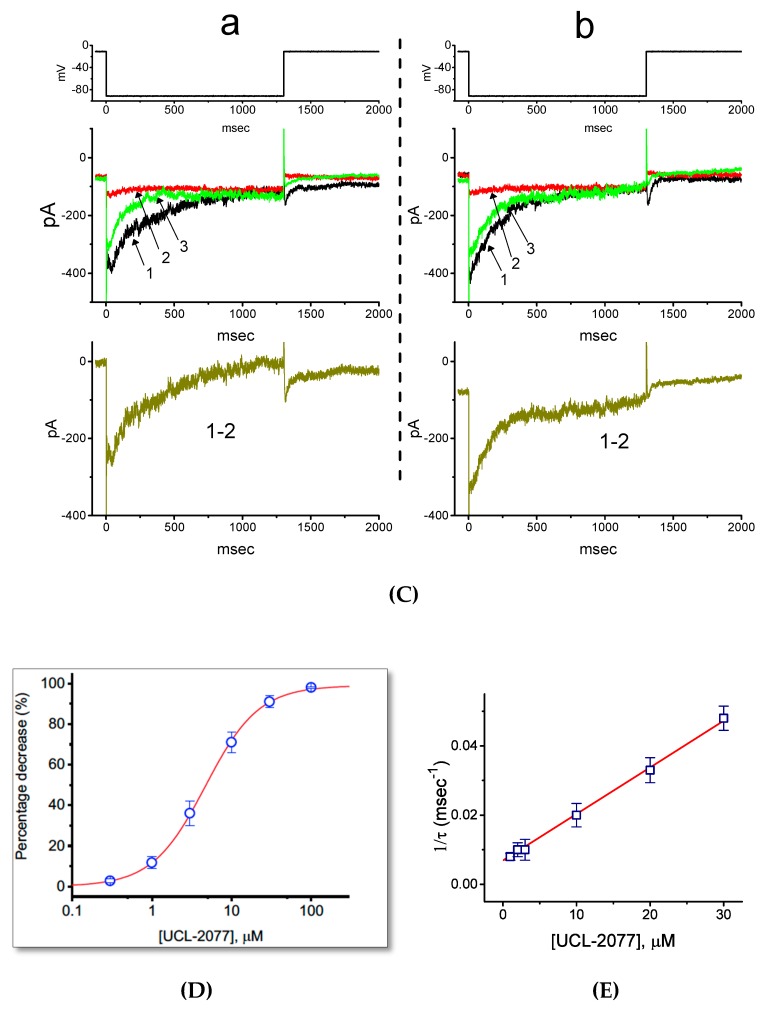
Effect of UCL-2077 on hyperpolarization-elicited *I*_K(erg)_ from pituitary tumor (GH_3_) cells. In these experiments, we bathed cells in a high-K^+^, Ca^2+^-free solution and we filled the recording pipette with K^+^-containing solution. (**A**) Representative current traces obtained in the control (a) and during cell exposure to 1 (b), 3 (c), 10 (d) or 100 μM (e) of UCL-2077. The upper part is the voltage protocol used for the elicitation of *I*_K(erg)_, while the arrowhead indicates the zero-current level. (**B**) *I*_K(erg)_ traces obtained in the control (a) and during exposure to 3 μM azimilide (b) or 10 μM aximilide (c). (**C**) *I*_K(erg)_ traces (upper part) obtained in the control (a1 and b1), during exposure to 10 μM azimilide (a2) or 10 μM E-4031 (b2), or after washout of each agent (a3 and b3), respectively. The lower panel indicates the azimilide- or E-4031-sensitive component (i.e., a1−a2 or b1−b2) of *I*_K(erg)_, respectively. The uppermost part in each panel is the voltage protocol used. (**D**) Concentration–response curve for UCL-2077-induced inhibition of *I*_K(erg)_ in these cells. The peak amplitude of *I*_K(erg)_ measured at the beginning of hyperpolarizing voltage step during exposure to UCL-2077 was compared with the control value (mean ± SEM, *n* = 8 for each point). The smooth line represents the best fit to the modified Hill equation (Equation (1)). The values for IC_50_, the Hill coefficient, and maximally inhibited percentage of *I*_K(erg)_ were 4.7 μM, 1.3, and 99%, respectively. In (**E**), the kinetics of a UCL-2077-induced block of *I*_K(erg)_ obtained from GH_3_ cells was assessed. The reciprocal of deactivating time constant (1/τ_deact_) of hyperpolarization-induced *I*_K(erg)_ obtained by exponential fits of the trajectory in decaying current was plotted as a function of the UCL-2077 concentration (mean ± SEM, *n* = 8 for each point). Data points were fitted by a linear regression (Equation (3)), indicating that there was a molecularity of 1 for UCL-2077-induced blocking. Blocking (*k*_+1_^*^) or unblocking (*k*_−1_) rate constants, derived from the slope and the *y*-axis intercept of the interpolated line, were computed to be 1.345 sec^−1^·μM^−1^ or 6.894 sec^−1^, respectively.

The whole-cell voltage-clamp experiments were initially applied to assess the effect of UCL-2077 on ionic currents in these cells. To record *I*_K(erg)_ and avoid the contamination of Ca^2+^-activated K^+^ currents, we suspended cells in high-K^+^, Ca^2+^-free solution, and the pipette was filled with K^+^-containing solution. When the cell was maintained at −10 mV and 1-sec hyperpolarizing voltage, pulses ranging between −10 and −90 mV were applied. As cells were exposed to different concentrations of UCL-2077, the *I*_K(erg)_ amplitude was progressively decreased and the deactivation time course of the current concomitantly became accelerated (Figure 1A). For example, upon hyperpolarization to −90 mV from a holding potential of −10 mV, UCL-2077 at a concentration of 10 μM produced a substantial reduction in the peak amplitude of *I*_K(erg)_ from 276 ± 13 to 109 ± 8 pA (*n* = 11, *p* < 0.05). After the washout of this compound, the current amplitude returned to 246 ± 11 (*n* = 9). The addition of either E-4031 (10 μM) or azimilide (10 μM) suppressed the amplitude of *I*_K(erg)_, while chlorotoxin (1 μM) failed to cause any effect on it. The inhibitory effect of azimilide on *I*_K(erg)_ is illustrated in Figure 1B. Moreover, the azimilide- or E-4031-sensitive component of *I*_K(erg)_ during long-lasting membrane hyperpolarization was depicted in Figure 1C. However, neither the further addition of diazoxide (30 μM) nor cilostazol (10 μM) was able to reverse UCL-2077-mediated inhibition of this current. Diazoxide or cilostazol are activators of ATP-sensitive K^+^ or BK_Ca_ channels [30], respectively, while chlorotoxin is a blocker of Cl^-^ channels.

We further determined the relationship between the UCL-2077 concentration and the percentage inhibition of *I*_K(erg)_. According to Equation (1), by virtue of a nonlinear least-squares fit to the results, the IC_50_ value required for the inhibitory effect of this compound on *I*_K(erg)_ in GH_3_ cells was calculated to be 4.7 μM, and, at a concentration of 100 μM, it almost completely suppressed current amplitude (Figure 1D). These experimental results reflect that UCL-2077 exerts a depressant action on hyperpolarization-evoked *I*_K(erg)_ in GH_3_ cells.

During cell exposure to UCL-2077, in addition to the depressed amplitude of *I*_K(erg)_, the deactivation time course of *I*_K(erg)_ elicited by large membrane hyperpolarization tended to be fastened. Therefore, in attempts to obtain more quantitative evidence for UCL-2077-induced blocking of *I*_K(erg)_, the time constants for *I*_K(erg)_ deactivation taken with or without addition of different concentrations of this compound were measured by appropriately fitting each deactivation trajectory of hyperpolarization-elicited current to a single exponential function. Based on the reaction scheme (Equations (2) and (3)), the relationship of 1/τ_deact_ versus [B] (i.e., UCL-2077 concentration) became linear (Figure 1E); hence, the blocking (*k*_+1_^*^) and unblocking (*k*_−1_) rate constants were estimated to be 1.345 sec^−1^μM^−1^ and 6.894 sec^−1^, respectively, which yielded a value of 5.1 μM for the dissociation constant (*K*_D_ = *k*_−1_/*k*_+1_^*^). Notably, this value is nearly close to the effective IC_50_ elaborated above.

**Figure 2:**
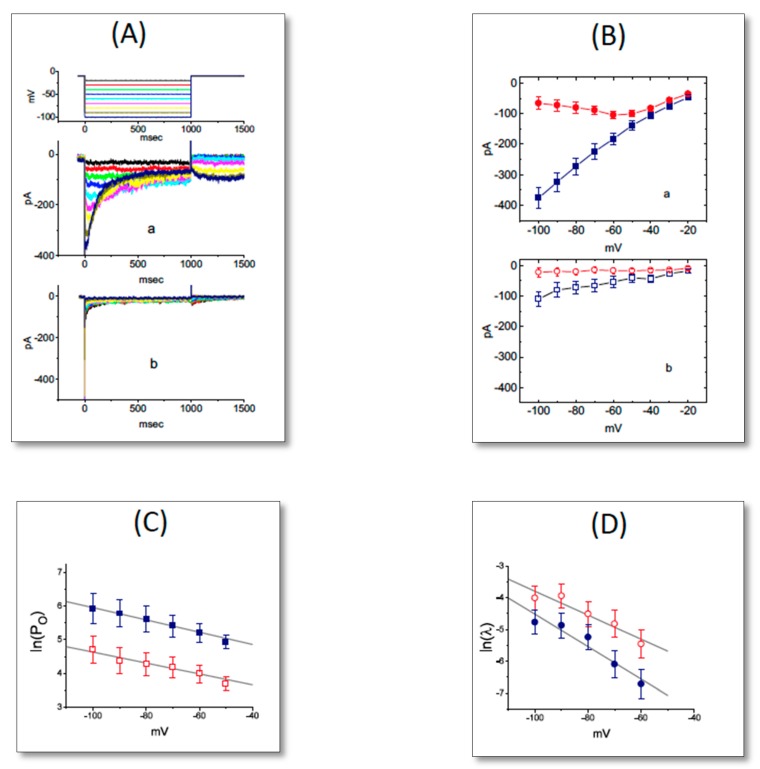
Effect of UCL-2077 on the *I-V* relationship of *I*_K(erg)_ in GH_3_ cells. In these experiments, we maintained each cell at −10 mV and the 1-sec hyperpolarizing command voltages ranging between −100 and −20 mV were delivered. (**A**) Superimposed current traces in the absence (a) or presence (b) of 30 μM UCL-2077. The voltage profile applied for current elicitation is indicated in the uppermost part of (A). (**B**) Averaged *I-V* relationships of *I*_K(erg)_ in the control (a, closed symbols) and during exposure to 30 μM UCL-2077 (b, open symbols). Current amplitudes in each panel were obtained at the beginning (square symbols) and end (circle symbols) of voltage pulses (mean ± SEM, *n* = 8 for each point). (**C**) Relationships of ln(P_O_) versus membrane potential in the control (■) and during cell exposure (□) to 30 μM UCL-2077. The P_O_ denotes macroscopic open probability and is reflected by the peak amplitude of *I*_K(erg)_ elicited by membrane hyperpolarization. Based on equation (4), the slopes of dashed lines for the absence or presence of 30 μM UCL-2077 were estimated to be −0.018 or −0.016, respectively. (**D**) Relationships of ln(*λ*) versus membrane potential (see equation (5)) obtained with or without addition of 30 μM UCL-2077. The slope indicated in straight lines for the absence (●) and presence (○) of 30 μM UCL-2077 was −0.051 or −0.038, respectively.

We further examined the effect of UCL-2077 on the *I-V* relationship of *I*_K(erg)_ in GH_3_ cells. Figure 2A illustrates original records obtained after applying 1-sec hyperpolarizing steps from a holding potential of −10 mV to membrane potentials between −100 and −20 mV in 10-mV steps taken with or without the addition of this compound. The magnitude of UCL-2077-mediated inhibitory effect on *I*_K(erg)_ measured at the beginning of hyperpolarizing pulses notably became higher than that at the end of voltage steps (Figure 2B). The presence of UCL-2077 (30 μM) led to a resultant decrease in the slope of linear fit of the peak *I*_K(erg)_ amplitudes to voltages between −100 and −60 mV from 4.88 ± 0.12 to 1.26 ± 0.06 nS (*n* = 8, *p* < 0.05). These results indicate that *I*_K(erg)_ remained functionally active in GH_3_ cells [15,18], and that its blocking effect was mainly exerted on the *I*_K(erg)_, which is responsible for inward rectification.

### 2.2. Effect of UCL-2077 on the Gating Charge of I_K(erg)_ Activation and Deactivation

Based on the limiting slope method described in Equation (4), we further performed the estimation of the gating charge (*z*_a_) involved in *I*_K(erg)_ activation. Under our experimental conditions, we applied this equation to create a lower bound to the total gating charge by approaching it in the limit of the negative potential applied. As illustrated in Figure 2C, the slope of the linear region ranging between −100 and −50 mV without or with the application of 30 μM UCL-2077 was derived and then estimated to be −0.018 or −0.016, respectively. As we applied those values to Equation (4), the *z*_a_ value taken in the control or during cell exposure to UCL-2077 was then computed to be 0.46 ± 0.02 or 0.40 ± 0.02 *e* (*n* = 8), respectively. Thus, it is clear that the *z*_a_ value used for *I*_K(erg)_ activation in the presence of UCL-2077 became significantly smaller (*p* < 0.05).

The presence of UCL-2077 tends to speed up the deactivation rate of *I*_K(erg)_ in response to long-lasting step command hyperpolarization. For this reason, we also further analyzed the effect of this compound on the *z*_deact_ value from GH_3_ cells. According to Equation (5), we estimated the *z*_deact_ values obtained with or without the addition of UCL-2077. As depicted in Figure 2D, we further constructed the relationships of ln(λ) versus membrane potential and the slopes of linear region between −100 and −60 mV in the control (i.e., UCL-2077 was not present) and during exposure to 30 μM UCL-2077 were thereafter noted to be −0.051 and −0.038, respectively. When we applied those values to Equation (5), the *z*_deact_ value in the control or during cell exposure to UCL-2077 was calculated to be 1.27 ± 0.03 or 0.94 ± 0.02 *e* (*n* = 9), respectively. During the exposure to UCL-2077, the *z*_deact_ value involved in *I*_K(erg)_ deactivation became significantly smaller (*p* < 0.05). The magnitude of decrease in *z*_deact_ (0.35 *e*) caused by UCL-2077 was much higher than that in *z*_a_ (0.06 *e*), reflecting that the action of UCL-2077 on *I*_K(erg)_ tended to be preferentially exerted on the deactivation process of K_erg_ channels.

### 2.3. Steady-State Activation of I_K(erg)_ Altered by the Presence of UCL-2077

**Figure 3:**
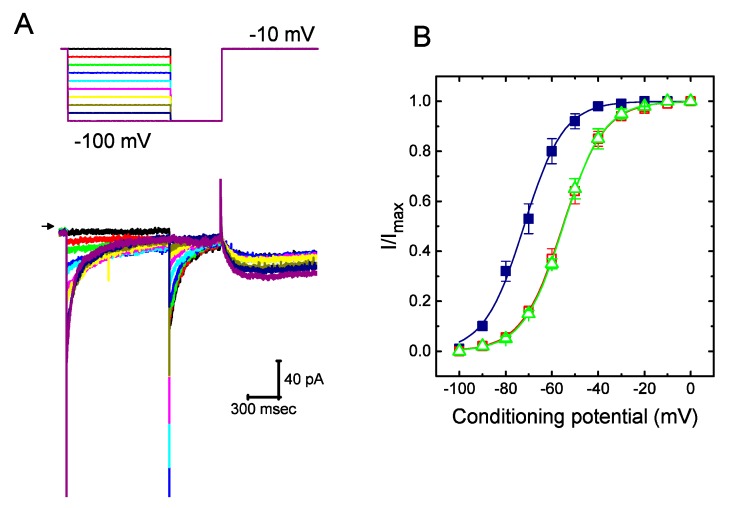
Effect of UCL-2077 on steady-state activation of *I*_K(erg)_ in GH_3_ cells. (**A**) Superimposed *I*_K(erg)_ traces obtained during cell exposure to UCL-2077 (10 μM). We bathed cells in high-K^+^, Ca^2+^-free solution and, during the recordings, filled the recording electrode by using K^+^-containing solution. The 1-sec conditioning hyperpolarizing pulses to various voltage steps ranging from −100 to 0 mV in 10-mV increments were applied to the examined cell from a holding potential of −10 mV, and following each conditioning pulse, a 500-msec test pulse to −100 mV was thereafter delivered to elicit *I*_K(erg)_. The upper part is the voltage protocol used and arrowhead indicates the zero current level. (**B**) Steady-state activation of *I*_K(erg)_ in the absence (■) and presence of 10 μM UCL-2077 (□) or 10 μM azimilide (△) (mean ± SEM; *n* = 9 for each point). The smooth lines were approximately fitted to Equation (6).

To further characterize the suppressive effect of UCL-2077 on *I*_K(erg)_, we next tested the voltage dependence of the effect of UCL-2077 on *I*_K(erg)_ in GH_3_ cells. Figure 3 shows the steady-state activation curve of *I*_K(erg)_ in the absence or presence of UCL-2077 (10 μM). In these experiments conducted with a two-step command voltage protocol, a 1-sec conditioning pulse to different voltages was delivered to precede the test pulse to −100 mV from a holding potential of −10 mV. We then constructed the relationship between the conditioning potentials and the normalized amplitudes of *I*_K(erg)_ and then fitted the data set with a Boltzmann function (see equation (6)). In the control, the voltage for half-maximal activation (*V*_1/2_) or the corresponding slope factor (*k*) was −72.1 ± 1.9 or 8.6 ± 0.9 mV(*n* = 9), respectively; in the presence of UCL-2077 (10 μM), *V*_1/2_ or *k* was −55.3 ± 1.8 or 8.9 ± 0.9 mV(*n* = 9), respectively; and in the presence of azimilide (10 μM), *V*_1/2_ or *k* was −55.1 ± 1.7 or 8.7 ± 0.9 mV(*n* = 9), respectively. It thus became notable that the addition of UCL-2077 (10 μM) distinctly shifted the midpoint of the activation curve along the voltage axis toward the depolarizing voltage by roughly 17 mV (*p* < 0.05) with no substantial change in the slope factor (i.e., *k* value) of the current. Strikingly, under our experimental conditions, the data showed that, in addition to the decreased *I*_K(erg)_ amplitude, there was a notable voltage dependence of the steady-state activation curve of this current in its presence.

### 2.4. Effect of UCL-2077 on the Voltage Hysteresis Elicited in Response to Triangular Ramp Pulse

**Figure 4:**
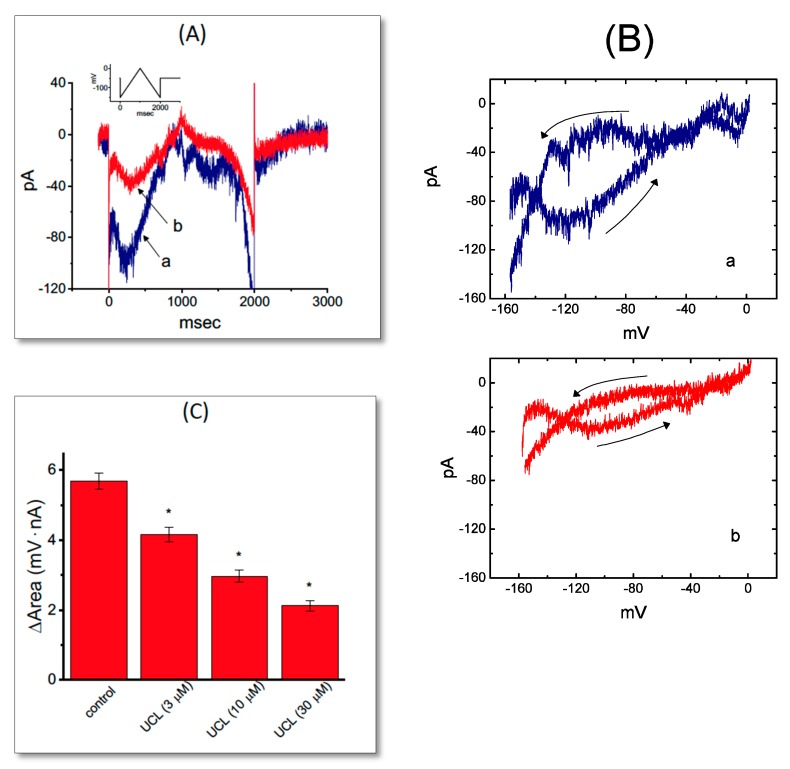
Effect of UCL-2077 on the voltage hysteresis of *I*_K(erg)_ identified from GH_3_ cells. (**A**) Original current trace elicited in response to 2-sec long-lasting triangular (i.e., upsloping and downsloping) ramp pulse between −150 and 0 mV. The inset in (**A**) shows the voltage protocol delivered. Current trace labeled a is control, while that labeled b was obtained in the presence of 10 μM UCL-2077. (**B**) Voltage hysteresis of *I*_K(erg)_ measured from the absence (a) or presence (b) of 10 μM UCL-2077. Arrows indicate the direction of *I*_K(erg)_ in which time passes. (**C**) Summary bar graph showing the effect of UCL-2077 on the ∆area of voltage hysteresis (mean ± SEM, *n* = 7 for each bar). ^*^ Significantly different from control (*p* < 0.05).

The voltage hysteresis of ionic currents has been demonstrated to have an impact on electrical behaviors of AP firing [31,32]. We thus explored whether there is possible voltage hysteresis existing in *I*_K(erg)_ measured from GH_3_ cells. In this set of experiments, a long-lasting triangular ramp pulse with a duration of 2 sec (i.e., ±0.15 V/sec) was delivered as the whole-cell configuration was firmly achieved. Interestingly, as illustrated in Figure 4A, the trajectories of *I*_K(erg)_ elicited by the upsloping (i.e., depolarized from −150 to 0 mV) and downsloping (hyperpolarized from 0 to −150 mV) ramp pulse as a function of time were distinguishable between them. The current amplitude elicited by the upsloping limb of the triangular voltage ramp was less than that by the downsloping limb, strongly indicating that there was a voltage hysteresis for this current, as depicted in Figure 4B (i.e., the relationship of current amplitude versus membrane potential). As the ramp speed was reduced, the hysteresis degree for *I*_K(erg)_ became progressively increased. Notably, as the examined cell was exposed to UCL-2077 (10 μM), the *I*_K(erg)_ amplitude evoked in the upsloping limb of long-lasting triangular ramp was found to be significantly decreased to a lesser extent than that measured from the downsloping limb. For example, in the controls, the *I*_K(erg)_ amplitudes at −120 mV elicited upon the upward and downward end of triangular ramp pulse were 35.6 ± 2.4 and 97.6 ± 4.2 pA (*n* = 7), respectively, and the values were found to differ significantly between them (*p* < 0.05). However, during exposure to UCL-2077 (10 μM), the amplitudes of forward and backward *I*_K(erg)_ measured at the same level of membrane potential were substantially reduced to 19.2 ± 1.9 and 39.5 ± 2.3 pA (*n* = 7, *p* < 0.05), respectively.

We next quantified the degree of voltage hysteresis on the basis of the difference in areas under the curve in the forward (upsloping) and reverse (downsloping) direction as indicated by the arrows in Figure 4B. It was seen that for *I*_K(erg)_ in GH_3_ cells, the degree of voltage hysteresis increased with slower ramp speed, and that the presence of UCL-2077 led to a conceivable reduction in the amount of such hysteresis. Figure 4C illustrates a summary of the data showing the effect of UCL at different concentrations on the areas under the curve between the forward and backward current traces. For example, the addition of UCL-2077 (10 μM) substantially decreased the area by about 50%, elicited by such a long-lasting triangular voltage ramp.

### 2.5. Inhibitory Effect of UCL-2077 on Delayed-Rectifier K^+^ Current (I_K(DR)_)

**Figure 5:**
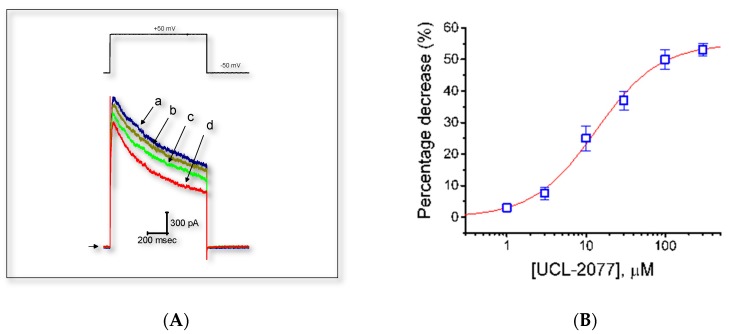
Effect of UCL-2077 on *I*_K(DR)_ in GH_3_ cells. In these experiments, we bathed cells in Ca^2+^-free Tyrode’s solution, which contained 1 μM TTX to suppress Na^+^ current, and backfilled the pipette with K^+^-containing solution. (**A**) Representative *I*_K(DR)_ traces elicited by step depolarization (indicated in the upper part) were obtained in the absence (a) and presence of 3 (b), 10 (c), or 30 μM (d) UCL-2077. (**B**) Concentration-response curve for UCL-2077-induced inhibition of *I*_K(__DR)_ in these cells. The peak amplitude of *I*_K(__DR)_ measured at the end of depolarizing voltage step during exposure to UCL-2077 was compared with the control value (mean ± SEM, *n* = 8 for each point). The smooth line represents the best fit to the modified Hill equation (equation (1)). The values for IC_50_, the Hill coefficient, and maximally inhibited percentage of *I*_K(__DR)_ were 13.4 μM, 1.1, and 55%, respectively.

We further examined another type of voltage-gated K^+^ current, namely *I*_K(DR)_, inherently present in GH_3_ cells with or without the addition of UCL-2077. To measure *I*_K(DR)_, we bathed cells in Ca^2+^-free Tyrode’s solution containing 1 μM tetrodotoxin (TTX), and the recording pipette was filled with K^+^-containing solution. As these cells were exposed to UCL-2077 at different concentrations, the amplitude of *I*_K(DR)_ evoked by 1-sec step depolarization from −50 to +50 mV was slightly but significantly suppressed (Figure 5A). The addition of UCL-2077 at a concentration of 1 μM was not found to have any effects on *I*_K(DR)_. However, as the membrane potential was depolarized from −50 to +50 mV, this compound (30 μM) significantly decreased the peak amplitude of *I*_K(DR)_ from 2117 ± 181 to 1741 ± 119 pA (*n* = 8, *p* < 0.05). Concomitant with this finding, the inactivation time constant of *I*_K(DR)_ in response to membrane depolarization was substantially shortened to 36.3 ± 1.3 msec (*n* = 7, *p* < 0.05) from a control value of 21.7 ± 1.1 msec (*n* = 7), when cells were exposed to 30 μM UCL-2077. We next assessed the relationship between the UCL-2077 concentration and the percentage inhibition of *I*_K(__DR)_. According to Equation (1), by virtue of a nonlinear least-squares fit to the results, the IC_50_ value required for the inhibitory effect of this compound on *I*_K(erg)_ in GH_3_ cells was calculated to be 13.4 μM (Figure 5B). By comparison to its effect on *I*_K(erg)_, the presence of UCL-2077 became less effective in suppressing *I*_K(DR)._

### 2.6. Failure of UCL-2077 to Alter the Activity of BK_Ca_ Channels

**Figure 6:**
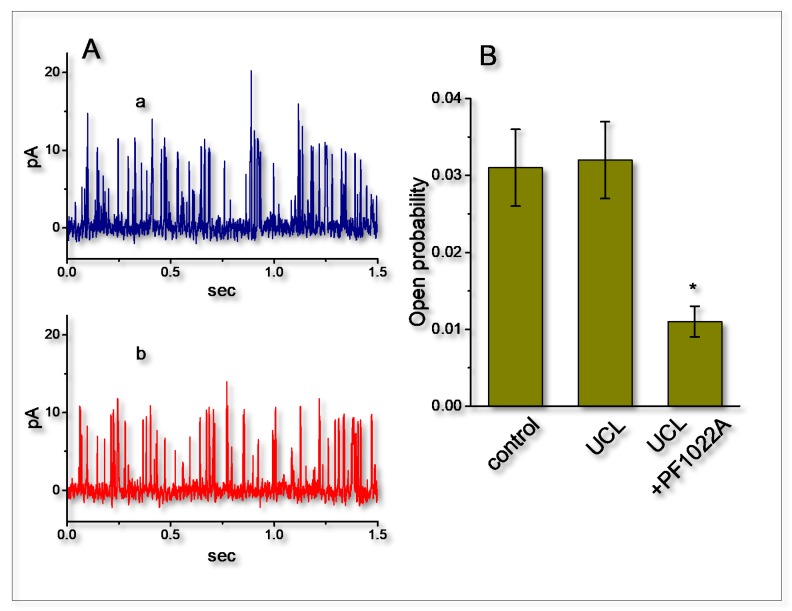
Failure of UCL-2077 to perturb the activity of BK_Ca_ channels functionally expressed in GH_3_ cells. In these experiments, inside-out current recordings were made, cells were bathed in high-K^+^, Ca^2+^-free solution, bath solution contained 0.1 μM Ca^2+^ and the examined cell was held at +60 mV. (**A**) Original BK_Ca_-channel currents obtained in the absence (a) and presence (b) of 10 μM UCL-2077. The upward deflection is the opening event of the channel. (**B**) Bar graph depicting effects of UCL-2077 (10 μM) and UCL-2077 (10 μM) plus PF1022A (10 μM) on the probabilities of BK_Ca_ channels that would be actively open (mean ± SEM, *n* = 8 for each bar). ^*^ denotes significant difference from control (*p* < 0.05).

The activity of BK_Ca_ channels (maxi-K^+^ channels, K_Ca_1.1, *KCNMA1*, *Slo1*) functionally expressed in excitable cells has been reported to be intimately linked to the afterhyperpolarization of neurons [3,9,33]. As such, we intended to examine whether the presence of UCL-2077 perturbed the activity of BK_Ca_ channels in GH_3_ cells. In these experiments, we conducted inside-out current recordings with symmetrical K^+^ solution (145 mM). The bath medium contained 0.1 μM Ca^2+^, and the potential was set at +60 mV. As shown in Figure 6, when we applied this compound at a concentration of 10 μM to the bath (i.e., cytoplasmic side of the excised patches), no measurable change in the probability of BK_Ca_-channel openings maintained at +60 mV (0.031 ± 0.005 (in the control) versus 0.032 ± 0.005 (in the presence of UCL-2077), *n* = 8, *p* > 0.05) was found. The single-channel conductance of BK_Ca_ channels did not differ significantly (168 ± 8 pS [in the control] versus 168 ± 7 pS [in the presence of 10 μM UCL-2077]; *n* = 8, *p* > 0.05). However, the further application of PF1022A (10 μM), still in continued presence of UCL-2077, was effective in suppressing the activity of BK_Ca_ channels without any changes in single-channel amplitude, as evidenced by a significant decrease in channel opening probability to 0.011 ± 0.002 (*n* = 8, *p* < 0.05). PF1022A, a nematocidal agent, has been reported to suppress the activity of BK_Ca_ channels [34]. However, it needs to be noted that a different substance (i.e., cyclooctadepsipeptide emodepside) was reported to activate the Slo-1 channel [34]. The reason for this difference in stimulatory or inhibitory actions on BK_Ca_ channels is currently unknown; therefore, it remains to be further studied. Nevertheless, unlike effects of UCL-2077 on *I*_K(erg)_ or *I*_K(DR)_, the data showed that there was minimal effect on the probability of BK_Ca_-channel openings observed in GH_3_ cells. 

### 2.7. Suppressive Effect of UCL-2077 on the Activity of IK_Ca_ Channels

**Figure 7:**
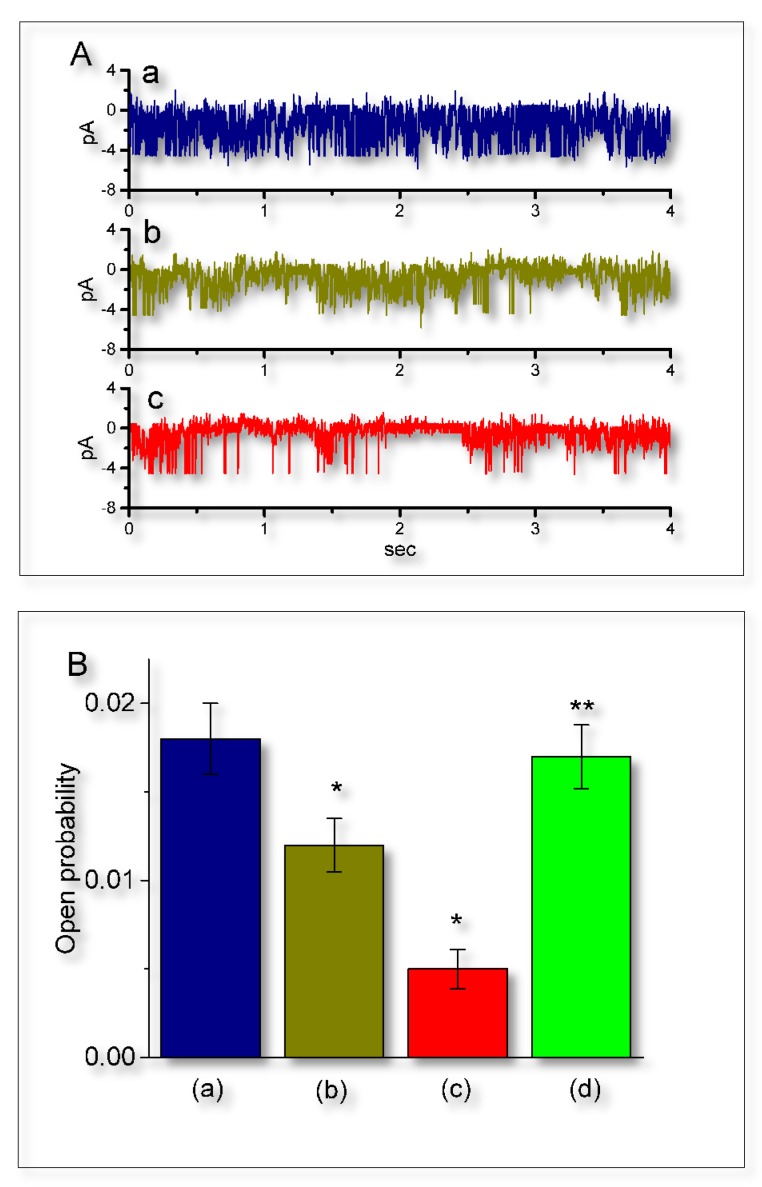
Suppressive effect of UCL-2077 on the open probability of IK_Ca_ channels in GH_3_ cells. Under these experimental conditions, we suspended cells in normal Tyrode’s solution containing 1.8 mM CaCl_2_. The recording pipette was backfilled with K^+^-containing solution and we then performed cell-attached current recordings. (**A**) Original IK_Ca_-channel currents obtained in the control (a) and after application of 3 (b) and 10 μM (c) UCL-2077. The potential was held at 0 mV relative to the bath. Notably, the downward deflection indicates channel openings. The time scale in the lower panel applies to all current trace. (**B**) Summary bar graph showing effects of UCL-2077 and UCL-2077 plus 9-phenanthrol on the channel opening probability of IK_Ca_ channels (mean ± SEM, *n* = 9 for each bar). a: control; b: 3 μM UCL-2077; c: 10 μM UCL-2077; and d: 10 μM UCL-2077 plus 10 μM 9-phenanthrol. In the experiments on UCL-2077 plus 9-phenanthrol, 9-phenanthrol (10 μM), in the continued presence of 10 μM UCL-2077, was further added to the bath. ^*^ Significantly different from control (*p* < 0.05) and ^**^ significantly different from UCL-2077 (10 μM) alone group (*p* < 0.05).

Earlier reports have demonstrated that the magnitude of IK_Ca_-channel activity could play essential roles in the occurrence of slow afterhyperpolarization appearing in endocrine cells, CA1 pyramidal neurons and enteric neurons [3,5,21,26,27]. Therefore, the possible effect of UCL-2077 on IK_Ca_ channels in GH_3_ cells was further explored. In these experiments, we immersed cells in normal Tyrode’s solution which contained 1.8 mM CaCl_2_, and we held the examined cell at the potential of 0 mV relative to the bath. In keeping with previous observations [21], the IK_Ca_-channel activity, which displayed rapid open-close transitions, was robustly detected in GH_3_ cells. However, the addition of UCL-2077 was able to decrease the probability of IK_Ca_-channel openings with no substantial change in unitary amplitude of the channel (Figure 7A). For example, the presence of this compound (10 μM) significantly decreased the channel opening probability from 0.018 ± 0.002 to 0.005 ± 0.001 (*n* = 9, *p* < 0.05). No change in the single-channel amplitude of IK_Ca_ channels between the absence and presence of 10 μM UCL-2077 was demonstrated (3.87 ± 0.03 pA (control) versus 3.85 ± 0.03 pA (in the presence of UCL-2077); *n* = 9, *p* > 0.05). In the continued presence of UCL-2077 (30 μM), further addition of 9-phenanthrol (10 μM), an activator of IK_Ca_ channels [35], significantly reversed the UCL-2077-mediated suppression of IK_Ca_ channels in these cells (Figure 7B). Therefore, in addition to its perturbation on the amplitude and gating of *I*_K(erg)_, UCL-2077 was capable of diminishing the probability that IK_Ca_ channel would be open.

### 2.8. Effect of UCL-2077 on the Frequency of Spontaneous Action Currents (ACs)

**Figure 8:**
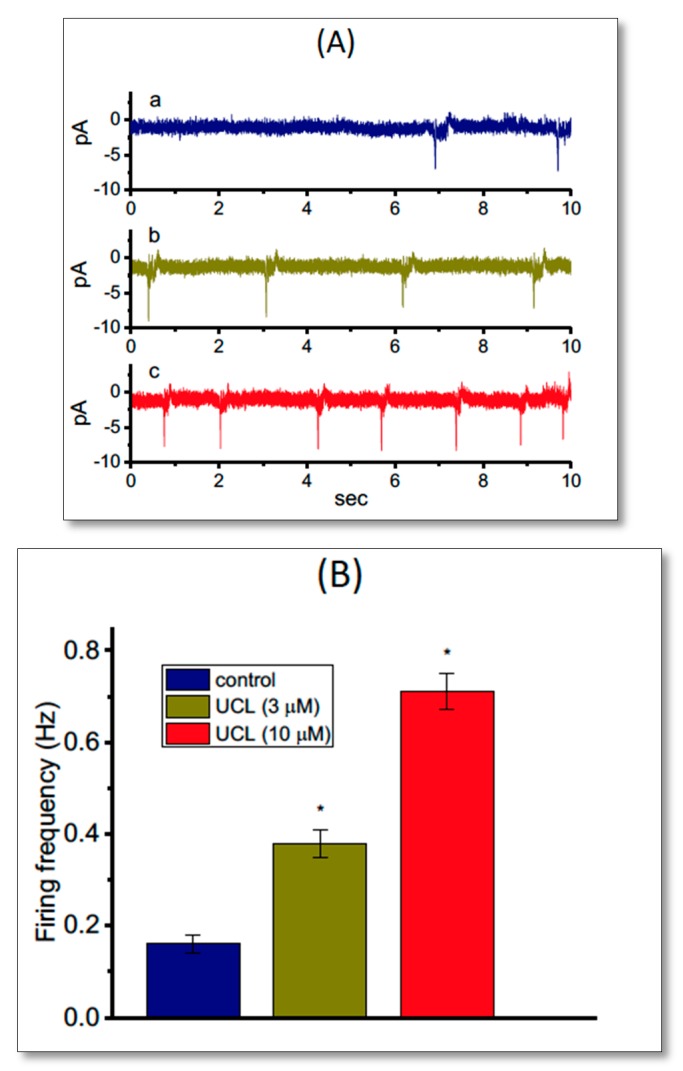
Stimulatory effect of UCL-2077 on action currents (ACs) identified from voltage-clamped GH_3_ cells. In these experiments, cells were bathed in normal Tyrode’s solution that contained 1.8 mM CaCl_2_, and cell-attached current recordings were done. The potential was maintained at the resting potential of a cell (−70 mV). (**A**) Original ACs obtained in the control (a) and during exposure to 3 μM (b) and 10 μM (c) UCL-2077. The inward deflection in each trace indicates the AC appearance. (**B**) Summary bar graph showing stimulatory effect of 3 or 10 μM UCL-2077 (UCL) on AC frequency of GH_3_ cells (mean ± SEM; *n* = 9 for each bar). ^*^ Significantly different from control (*p* < 0.05).

In another set of recordings, we investigated whether UCL-2077 modulated the frequency of spontaneous ACs measured from GH_3_ cells. In these cell-attached voltage-clamp recordings, we bathed cells in normal Tyrode’s solution containing 1.8 mM CaCl_2_, and we maintained the potential at the resting potential of the cell. As depicted in Figure 8, the addition of UCL-2077 increased the AC frequency significantly; however, it did not alter the AC amplitude significantly. For example, as cells were exposed to UCL-2077 (10 μM), the firing frequency of ACs seen in GH_3_ cells was significantly raised to 0.71 ± 0.004 Hz (*n* = 9, *p* < 0.05) from a control value of 0.16 ± 0.002 Hz (*n* = 9).

We then also examined whether UCL-2077 had any effects on AC firing in GH_3_ cells directed against *KCNH2*. When we knocked this down in these cells, the frequency of ACs recorded from cell-attached current recordings was not changed in the presence of 10 μM UCL-2077 (0.71 ± 0.004 Hz (in the control) versus 0.69 ± 0.004 Hz (in the presence of 10 μM UCL-2077), *n* = 7, *p* > 0.05). In contrast, in cells transfected with a scrambled negative control of siRNA, the presence of 10 μM UCL-2077 increased AC frequency from 0.21 ± 0.002 Hz to 0.72 ± 0.004 Hz (*n* = 7, *p* < 0.05). Therefore, changes in AC frequency caused by this compound are explained by its modifications on K_erg_ channels.

## 3. Discussion

The present results showing that UCL-2077 tended to accelerate *I*_K(erg)_ deactivation suggest that the UCL-2077 molecule might reach the blocking site only when the channel remains functionally active in the open state. UCL-2077 likely has a higher affinity toward the open-deactivated K_erg_ channels than toward the closed (or resting) channels in GH_3_ cells. Another pertinent finding in this study is that, based on the estimation of changes in the *z*_a_ and *z*_deact_ values required for *I*_K(erg)_ elicitation (Equations (4) And (5)), the addition of UCL-2077 significantly allowed those values to be smaller and the magnitude of its decrease in *z*_deact_ was greater than that in *z*_a_. Our data suggest that, in addition to its inhibition of *KCNQ* channels [4], the UCL-2077 molecule may interact largely with the deactivation process to alter both the magnitude and kinetics of *I*_K(erg)_. 

In our observations, the concentration-dependent inhibition of *I*_K(erg)_ with effective IC_50_ of 4.7 μM was depicted in the presence of UCL-2077. This value is nearly close to the *K*_D_ value (5.1 μM), taken according to the first-order reaction scheme (Equations (2) and (3)). It was also noted that the values of IC_50_ or *K*_D_ appear to be higher than used for its suppression of KCNQ1- or KCNQ2-encoded currents as reported previously [4]. However, the presence of this compound was found to distinctly shift the steady-state activation curve of *I*_K(__erg)_ along the voltage axis to a less hyperpolarized potential, as well as reducing the magnitude of voltage hysteresis for *I*_K(erg)_ in response to a long-lasting triangular ramp pulse. Under cell-attached current recordings, it substantially increased the AC frequency in a concentration-dependent manner. As such, K_erg_ channels functionally expressed in electrically excitable cells are likely to be a viable target for its action, regardless of the detailed ionic mechanisms.

In our study, the probability of BK_Ca_-channel openings in GH_3_ cells was barely affected by the presence of UCL-2077. However, the magnitude of IK_Ca_-channel openness was clearly subject to reduction by this compound, despite the failure to reduce the unitary amplitude. Moreover, the UCL-2077-mediated decrease in IK_Ca_-channel activity was notably counteracted by the subsequent application of 9-phenanthrol, an activator of IK_Ca_ channels [35]. Previous observations have shown that the magnitude of IK_Ca_-channel activity could have significant impacts on the slow afterhyperpolarization of neurons [21,26,27,29]. Therefore, UCL-2077-mediated actions on the electrical behaviors of neurons or endocrine cells may partly ascribe from its reduction in the probability of IK_Ca_ channels that would be open [3].

Voltage-hysteresis has been proposed to play a role in influencing the electrical behavior of excitable cells. In this study, consistent with previous observations in HCN channels [31,32], K_erg_ channels inherently existing in GH_3_ cells were found to undergo either a hysteresis in their voltage-dependence, or a mode-shift, in which the voltage sensitivity of gating charge movements depends on the previous state. In the current study, we also explored the perturbating effect of UCL-2077 on such non-equilibrium property inherently in K_erg_ channels. Our results clearly show that the presence of this compound was able to decrease the *z*_a_ and *z*_deact_ values required for the elicitation of *I*_K(erg)_ by membrane hyperpolarization. Therefore, the detailed studies regarding whether UCL-2077 can act on a deactivated process during the elicitation of K_V_11-encoded channels and modify the heart function remain to be further delineated. Although the underlying mechanism of neuronal slow afterhyperpolarization is still unclear, previous studies have demonstrated the ability of UCL-2077 to modify slow after-hyperpolarization (AHP) [9,26] could be explained by its suppression of *I*_K(erg)_ and IK_Ca_-channel activity.

## 4. Materials and Methods

### 4.1. Drugs and Solutions

UCL-2077 (3-(triphenylmethylaminomethyl)pyridine; N-trityl-3-pyridinemethanamine; C₂₅H₂₂N₂; https://pubchem.ncbi.nlm.nih.gov/compound/24868317), cilostazol and diazoxide were acquired from Tocris (Union Biomed Inc., Taipei, Taiwan), and E-4031 was from Enzo (Farmingdale, NY, USA), while PF1022A was from MedChem (Princeton, NJ, USA), and 9-phenanthrol and tetrodotoxin (TTX) were from Sigma Chemical Co. (Merck Ltd., Taipei, Taiwan). Chlorotoxin was kindly acquired from Dr. Woei-Jer Chuang (Department of Biochemistry, National Cheng Kung University Medical College, Tainan, Taiwan), while azimilide was a gift from Procter and Gamble Pharmaceuticals (Cincinnati, OH, USA). Unless specified otherwise, culture media, penicillin-streptomycin and trypsin/EDTA were obtained from HyClone^TM^ (Thermo Fisher Scientific, Logan, UT, USA), while all other chemicals were of laboratory grade and were taken from standard sources. We used twice-distilled water that had been deionized through a Millipore-Q system (Millipore, Bedford, MA, USA) in all experiments.

The composition of the bath solution (i.e., standard HEPES-buffered normal Tyrode’s solution) in this study was 136.5 mM NaCl, 5.4 mM KCl, 5.4 mM KCl, 1.8 mM CaCl_2_, 0.53 mM MgCl_2_, 5.5 mM glucose, and 5.4 mM HEPES titrated to pH 7.4 with NaOH. To record *I*_K(DR)_, we backfilled the recording electrodes with a solution consisting of 130 mM K-aspartate, 20 mM KCl, 1 mM KH_2_PO_4_, 1 mM MgCl_2_, 3 mM Na_2_ATP, 0.1 mM Na_2_GTP, 0.1 mM EGTA, and 5 mM HEPES titrated to pH 7.2 with KOH; and, to measure *I*_K(erg)_, we replaced the bath solution with a high-K^+^, Ca^2+^-free solution consisting of 130 mM KCl, 10 mM NaCl, 3 mM MgCl_2_, 6 mM glucose, and 10 mM HEPES titrated to pH 7.4 with KOH. For the recording of BK_Ca_-channel activity, we used high-K^+^ bathing solution containing 145 mM KCl, 0.53 mM MgCl_2_, and 5 mM HEPES titrated to pH 7.4 with KOH, and we used the internal solution containing 145 mM KCl, 2 mM MgCl_2_ and 5 mM HEPES adjusted to pH 7.2 with KOH, while the value of free Ca^2+^ concentration was calculated assuming a dissociation constant for EGTA and Ca^2+^ (at pH 7.2) of 0.1 μM. For example, to provide 0.1 μM Ca^2+^ in the bath solution, 0.5 mM CaCl_2_ and 1 mM EGTA were added.

### 4.2. Cell Preparations

Pituitary GH_3_ cells, acquired from the Biorescources Collection and Research Center (BCRC-60015; Hsinchu, Taiwan), were routinely grown in Ham’s F12 medium, which was supplemented with 15% horse serum, 2.5% fetal calf serum, and 2 mM L-glutamine in a humidified environment of 5% CO_2_/95% air [18]. We commonly changed culture medium every 2–3 days for the removal of non-adhering cells, and cells would undergo passage as they reached confluence. To observe growth, we commonly used a Nikon Eclipse Ti-E inverted microscope (Li Trading Co., Taipei, Taiwan) which was equipped with a 5-megapixel cooled digital camera.

### 4.3. Transfection with siRNAs

Both Custom Stealth RNAi for rat *KCNH2* (sense: 5′-GACCUGCUUACUGCCCUCUACUUCA-3′, antisense: 5′-UGAAGUAGAGGGCAGUAAGCCAGGUC-3′) and a scrambled negative control of siRNA, which does not interfere with any known siRNAs, were acquired from Invitrogen (Carlsbad, CA, USA). *I*_K(erg)_ is encoded by *KCNH2*, which constitutes the pore-forming α-subunit of K_erg_ (or K_V_11.1) channels. In accordance with the protocol provided by the manufacturer, we transfected GH_3_ cells with 25 nM of siRNAs using a transfection reagent (Lipofectamine RNAiMAX; Invitrogen). We used small concentrations of siRNAs to prevent both off-target effects and any nonspecific responses to them. Gene-specific siRNA oligomers were diluted in reduced serum medium (Opti-MEM I; Invitrogen) and were subsequently mixed with a transfection reagent. As the complexes were incubated at room temperature for 20 min, they were thereafter added to the cells for another 24–48 h. We used electrophysiological recordings to evaluate the siRNA knockdown efficiency.

### 4.4. Electrophysiological Measurements

Shortly before the recordings were prepared, GH_3_ or NSC-34 cells were harvested with 1% trypsin/EDTA solution and an aliquot of cell suspension was thereafter transferred to a home-made recording chamber which was firmly mounted on the stage of an inverted DM-IL microscope (Leica Microsystems, Wetzlar, Germany). The examined cells were suspended at room temperature (20–25 °C) in HEPES-buffered normal Tyrode’s solution, the solution of which is elaborated above. To fabricate the patch electrodes from Kimax-51 capillaries (#34500; Kimble, Vineland, NJ, USA), we used either a PP-83 vertical puller (Narishige, Tokyo, Japan) or a P-97 Flaming/Brown programmable puller (Sutter, Novato, CA, USA), and we then fire-polished the tips with an MF-83 microforge (Narishige). During the measurements, the electrodes, which bore resistances between 3 and 5 MΩ when filled with different internal solutions, were maneuvered by the use of an MX-4 manipulator (Narishige) and precisely delicately operated by an MHW-3 hydraulic micromanipulator (Narishige). We performed patch-clamp recordings in whole-cell, cell-attached, or inside-out configuration by using either an RK-400 (Bio-Logic, Claix, France) or Axopatch-200B (Molecular Devices, Sunnyvale, CA, USA) amplifier [13,18]. Liquid junction potentials, which commonly develop at electrode tip when the composition of the internal solution was distinct from that in the bath, were nulled shortly before the seal formation was made, and whole-cell data were corrected by such junction potentials. The tested compounds were applied through perfusion or were added to the bath to achieve the final concentration indicated.

Action currents (ACs), which represent action potentials (APs), were achieved in the cell-attached current recordings [15,36,37]. For AC measurements, the potential was held at the level of the resting potential (~−70 mV). AC measurements were made to enable the quantification of the underlying firing frequency with no significant changes in intracellular milieu. We also assessed the frequency and amplitude of ACs recorded from GH_3_ or NSC-34 cells by using Mini Analysis Program (Synaptosoft, Leonia, NJ, USA).

### 4.5. Data Recordings and Analyses

The data, including voltage and current tracings, were simultaneously monitored and thereafter digitally stored in a TravelMate-6253 laptop computer (Acer, Taipei, Taiwan) at 10 kHz through a Digidata-1322A acquisition system (Molecular Devices). The device was equipped with an Adaptec Slim SCSI card (Milpitas, CA, USA) through the PCMCIA slot and, during the recordings, it was controlled by pCLAMP 9.2 (Molecular Devices). The current signals collected were low-pass filtered at 1 or 3 kHz to minimize electrical noise. Through digital-to-analog conversion, the pCLAMP-generated voltage profiles containing different types of rectangular or ramp waveform were particularly designed to assess the current-voltage (*I-V*) relationships, steady-state activation curve, and voltage hysteresis of ionic currents specified. If necessary for further analyses, the digitized signals were subsequently exported, either to 64-bit OriginPro 2016 (OriginLab, Northampton, MA, USA) or to the spreadsheets of Microsoft Excel (Redmond, WA, USA).

To calculate the percentage decrease in the peak amplitude of *I*_K(erg)_ obtained with or without the addition of UCL-2077, each cell was held at −10 mV and the hyperpolarizing potential to −90 mV with a duration of 1 sec was applied, while to determine the percentage decrease in *I*_K(DR)_ amplitude, each cell was held at −50 mV and the 1-sec depolarizing potential to +50 mV was applied. The peak amplitudes of hyperpolarization-induced *I*_K(erg)_ measured during cell exposure to different concentrations (0.3–100 μM) of UCL-2077 were compared with those measured after subsequent addition of E-4031 (10 μM). To evaluate the concentration-dependent relation of UCL-2077 on *I*_K(erg)_ or *I*_K(DR)_ amplitude, we fitted the data sets to the modified form of the Hill equation by using nonlinear regression analysis. That is:

(1) $$\%\;\mathrm{decrease}=\left(E_{\max}\times {\left[\mathrm{UCL}\right]}^{n_{H}}\right)/\left({\left[\mathrm{UCL}\right]}^{n_{H}}+{\mathrm{IC}}_{50}^{n_{H}}\right)$$

where [UCL] is the concentration of UCL-2077 applied; IC_50_ and n_H_ are the concentration needed to have a 50% inhibition and the Hill coefficient, respectively; and *E*_max_ is the UCL-2077-induced maximal reduction in the peak amplitude of *I*_K(erg)_ or *I*_K(DR)_ during hyperpolarizing or depolarizing steps.

The UCL-2077-mediated inhibition of *I*_K(erg)_ observed in GH_3_ cells can be predicted by a state-dependent blocking mechanism, in which the UCL-2077 molecule preferentially binds to the opening state of the channel on the basis of minimal first-order scheme [15]:

(2) $$C\;\begin{array}{c} \alpha \\ ⇄\\ \beta \end{array}\;O\;\begin{array}{c} k_{+1}^{*}.\left[B\right]\\ ⇄\\ k_{-1} \end{array}\;O\cdot B$$

where α or β denotes voltage-dependent rate constants for the opening or closing of K_erg_ channels; *k*_+1_^*^ or *k*_−1_ is blocking (forward) or unblocking (reverse) rate constants; and [B] is the UCL-2077 concentration. C, O or O·B is the closed, open or open-blocked states, respectively.

The blocking and unblocking rate constants were determined from the deactivation time constants (τ_deact_) of *I*_K(erg)_ during hyperpolarized steps, as the cells were exposed to different concentrations of UCL-2077. The deactivation time constants (τ_deact_) of *I*_K(erg)_ were calculated by fitting each current-trace trajectory to a single-exponential function. The apparent rate constants for binding (*k*_+1_^*^) and unbinding (*k*_−1_) were achieved from the following equation:

(3) $$\frac{1}{\tau_{deact}}={k_{+1}}^{*}\times [B]+k_{-1}$$

Specifically, the values of *k*_+1_^*^ and *k*_−1_ are estimated from the fit of 1/τ_deact_ versus different UCL-2077 concentration. Based on *k*_+1_^*^ and *k*_−1_, we were able to yield the value of dissociation constant (*K*_D_ = *k*_−1_/*k*_+1_^*^).

To determine the number of equivalent gating charges (*z*_a_) involved in *I*_K(erg)_ activation obtained with or without UCL-2077 addition, a less model-dependent “limiting slope” method [38,39] was used. That is,

(4) $$z_{a}=\underset{P_{O}\to 0}{\lim}k_{b}\cdot T\cdot \frac{d\ln(P_{O})}{dV}$$

where *z*_a_ is equivalent gating charge (in *e*) transferred during the K_erg_-channel activation; dln(P_O_)/d*V* is the slope of relationship between ln(P_O_) and testing potential *V*; and *k*_b_ and *T* are the Boltzmann constant and absolute temperature in Kelvin, respectively. This equation can provide a lower bound of the total gating charge, hence approaching it in the limit of very negative potential. This method was previously described to apply for a sequential model with one open state, where the transition rates are exponentially dependent on the voltage [38,39]. 

A linear regression analysis of the reciprocal of τ_deact_ (i.e., λ) versus V_test_ obtained with or without the addition of UCL-2077 was approximately implemented to yield an estimate of the gating charge (*z*_deact_) involved in the deactivation process according to the following equation:

$$\lambda_{V}=\lambda_{0}\cdot e^{\left[Z_{i}\cdot V\cdot \left(\frac{F}{RT}\right)\right]}$$

or

(5) $$\ln\left(\lambda_{V}\right)=\ln\left(\lambda_{0}\right)+Z_{i}\cdot V\cdot \left(\frac{F}{RT}\right)$$

where *λ*_V_ and *λ*_0_ correspond to the rate constants of deactivation (i.e., 1/τ_deact_) at voltage *V*_test_ and 0 mV, respectively, and *F*, *R* and *T* are Faraday’s constant, the universal gas constant and the absolute temperature, respectively. 

To construct the effect of UCL-2077 on the steady-state activation of *I*_K(erg)_ identified in GH_3_, the relationship of the conditioning potential versus the normalized amplitude of *I*_K(erg)_ derived with or without the UCL-2077 (10 μM) addition was elaborated as a Boltzmann function, as indicated in the following form:

(6) $$I=\frac{I_{\max}}{1+e^{\left(\frac{-(V-V_{1/2})}{k}\right)}}$$

where *I*_max_ denotes the maximal activated *I*_K(erg)_, *V* is the voltage in mV, *V*_1/2_ the voltage required for a half-maximal activation, and *k* the slope factor appearing in the inactivation curve for *I*_K(erg)_.

### 4.6. Single-Channel Analyses

Unitary current events of BK_Ca_ or IK_Ca_ channels digitized were evaluated by pCLAMP software (Molecular Devices). Multi-gaussian adjustments of the amplitude distributions occurring among channel events were applied to precisely determine the opening event of single channels. The functional independence among the channels was achieved as the observed stationary probabilities were compared. We evaluated the channel opening probabilities by using an iterative process to minimize the χ^2^ values calculated from a sufficiently large number of independent observations.

### 4.7. Statistical Analyses

The linear or nonlinear fitting to data sets (e.g., Hill and Boltzmann functions, and different time courses of *I*_K(erg)_ deactivation) was performed with the goodness of fit by virtue of different methods that include the Solver add-in function embedded inside Microsoft Excel 2013 (Redmond, WA, USA) and OriginPro 2016 (OriginLab). The results of the experiments are expressed as the mean ± standard error of the mean (SEM), in which sample sizes (*n*) are the cell number from which the data in separate set of experiments were achieved. The paired or unpaired *t* test followed by one-way ANOVA, together with the least-significance difference method for multiple-group comparisons among means, were implemented for statistical evaluation. However, if the Shapiro–Wilk normality test showed that the data were not normally distributed, a Kruskal–Wallis H test followed by Dunn’s multiple comparison tests would be done. The analyses were performed using SPSS version 20.0 (IBM, Armonk, NY, USA). The differences between values were considered significant when *p* < 0.05, unless stated otherwise.

## 5. Conclusions

The findings from the present study are as follows: (a) in pituitary GH_3_ cells, UCL-2077 decreases *I*_K(erg)_ in a dose- and state-dependent manner; (b) UCL-2077 decrease the z_a_ and z_deact_ values required for *I*_K(erg)_ elicitation; (c) this compound attenuates the voltage hysteresis of *I*_K(erg)_ evoked in response to a long-lasting triangular ramp pulse; (d) its presence suppressed the activity of IK_Ca_ channels; (e), under cell-attached current recordings, it decreased AC frequency. Therefore, these results strongly indicate that the convergent suppression by UCL-2077 of K_erg_ and IK_Ca_ channels may be responsible for its effects on the functional activities in these cells.

## Abbreviations

AC	action current
AP	action potential
BK_Ca_ channel	large-conductance Ca^2+^-activated K^+^ channel
*Erg*	*ether*-*à*-*go*-*go*-related gene
HH model	Hodgkin–Huxley model
*I*_K(DR)_	delayed rectifier K^+^ current
*I*_K(erg)_	*erg*-mediated K^+^ current
*I-V*	current versus voltage
IC_50_	the concentration required for a 50% inhibition
IK_Ca_ channel	intermediate-conductance Ca^2+^-activated K^+^ channel
*I*_K(DR)_	delayed-rectifier K^+^ current
*K*_D_	dissociation constant
K_erg_ channel	*erg*-mediated K^+^ channel
Λ	rate constant
SEM	standard error of mean
siRNA	short-interfering RNA
TTX	tetrodotoxin
*z*_a_	gating charge for *I*_K(erg)_ activation
*z*_αα_	gating charge for αα transition rate embedded in modeled K_erg_ channel
*z*_deact_	gating charge for *I*_K(erg)_ deactivation
